# Appraisal of WHO Guidelines in Maternal Health Using the AGREE II Assessment Tool

**DOI:** 10.1371/journal.pone.0038891

**Published:** 2012-08-13

**Authors:** Stephanie Polus, Priya Lerberg, Joshua Vogel, Kanokwaroon Watananirun, Joao Paulo Souza, Matthews Mathai, A. Metin Gülmezoglu

**Affiliations:** 1 Institute for Medical Informatics, Biometry and Epidemiology, University of Munich, Munich, Bavaria, Germany; 2 UNDP/UNFPA/WHO/World Bank Special Programme of Research, Development and Research Training in Human Reproduction (HRP), Department of Reproductive Health and Research, World Health Organization, Geneva, Switzerland; 3 Department of Community Medicine, University of Oslo, Oslo, Norway; 4 School of Population Health, University of Western Australia, Perth, Western Australia, Australia; 5 Faculty of Medicine, Siriraj Hospital, Mahidol University, Bangkok, Thailand; 6 Department of Maternal, Newborn, Child and Adolescent Health, World Health Organization, Geneva, Switzerland; Liverpool School of Tropical Medicine, United Kingdom

## Abstract

In 2007, the World Health Organization (WHO) received a criticism for a lack of transparency and systematic methods in the development of guidelines, which were at that time perceived as substantially driven by expert opinion. In this paper we assessed the quality of maternal and perinatal health guidelines developed since then. We used the Appraisal of Guidelines for Research and Evaluation (AGREE) II tool to evaluate the quality of methodological rigour and transparency of four different WHO guidelines published between 2007 and 2011. Our findings showed high scores among the most recent guidelines on maternal and perinatal health suggesting higher quality. However, there is still potential for improvement, especially in including different stakeholder views, transparency of guidelines regarding the role of the funding body and presentation of the guideline document.

## Introduction

The World Health Organization (WHO) has an important role in the provision of global guidance on health interventions and health care [1]. Developing evidence-based recommendations for a global audience enables informed decisions about clinical and programmatic interventions, public health actions and government policies [2]. During recent years, WHO has undergone significant procedural changes in guideline development methodology.

In 2007, Oxman, Lavis and Fretheim examined the use of evidence in WHO guidelines by interviewing senior staff. They found infrequent use of systematic reviews, an absence of a systematic guideline development methodology and a tendency to rely on expert opinion [3], despite evidence of the limitations of such an approach [4]–[9]. The study indicated poor internal support for guideline development, an absence of timelines for updating and lack of plans for dissemination and implementation of recommendations. Harm/benefit and cost analyses were rarely conducted or systematically reported. Guidelines were also sometimes difficult to identify due to different labelling such as ‘technical consultation’ or ‘report of a meeting’ [3].

The WHO Guideline Review Committee (GRC) was subsequently established “to ensure that WHO guidelines are of a high methodological quality and are developed through a transparent, evidence-based decision-making process” [10]. The WHO guideline handbook was revised and standards for reporting, processes, and evidence were established. WHO guideline development now follows a standardized process: (i) identification of questions related to clinical practice and health policy for which answers were needed; (ii) retrieval of up-to-date research-based evidence; (iii) assessment and synthesis of the evidence; (iv) formulation of recommendations with inputs from a wide range of stakeholders; and (v) formulation of plans for dissemination, implementation, impact evaluation and updating [11].

The WHO Department of Reproductive Health and Research (RHR) hosts the UNDP/UNFPA/WHO/World Bank Special Programme of Research, Development and Research Training in Human Reproduction (HRP). This programme is the main body within the United Nations system for research in sexual and reproductive health. RHR is responsible for normative guidance on sexual and reproductive health and rights [12]. RHR published the following maternal and perinatal health guidelines between 2007 and 2011: WHO recommendations for the prevention of postpartum haemorrhage (2007) (herein referred to as P-PPH), WHO guidelines for the management of PPH and retained placenta (2009, M-PPH), WHO recommendations for induction of labour (2011, IOL) and WHO recommendations for prevention and treatment of pre-eclampsia and eclampsia (2011, PE/E) [13]–[16]. The P-PPH guideline precedes the establishment of the WHO Guidelines Review Committee (GRC) and laid the foundation for the standards that followed [17].

This analysis aimed to assess the quality of maternal and perinatal health guideline development by examining the four guidelines described above. We used the Appraisal of Guidelines for Research and Evaluation (AGREE) II tool to evaluate each guideline [18]. The AGREE II tool is a widely-used instrument to assess methodological rigour and transparency of guideline development and has been tested for its validity and reliability. It uses a detailed framework to assess guideline quality, but also provides a methodological strategy for guideline development and content [18], [19]. The AGREE tool was recently used by the Reproductive Health Library (RHL) at WHO to appraise the quality of the P-PPH, M-PPH and IOL guidelines in a separate analysis. RHL engaged independent, external commentators with a strong obstetric background to conduct those appraisals [20].

## Methods

Four appraisers who had not previously participated in the development of WHO guidelines conducted the assessment (SP, PL, JV, KW). The appraisers used the online training tools recommended by the AGREE collaboration before conducting appraisals. Each of the four guidelines was rated independently with the AGREE II tool online by each appraiser. Appraisers did not communicate or confer with each other during the appraisal process.

The AGREE II tool encompasses 23 items in six domains: scope and purpose (3 items), stakeholder involvement (3 items), rigour of development (8 items), clarity of presentation (3 items), applicability (4 items), and editorial independence (2 items). The domain *scope and purpose* assesses whether the guideline describes its overall objective and target population clearly. A guideline should entail a clear definition of the target users, as well as demonstrate that the views and preferences of the target population (e.g. patients, public) have been sought and that the guideline development group includes all relevant professional groups which is assessed in *stakeholder improvement*. The next domain assesses the rigour of guideline development, encompassing systematic literature searching methodology, transparency of evidence-gathering process and whether evidence is explicitly linked to the recommendations. It also asks if the guideline has been externally reviewed prior to publication and if an updating strategy has been documented. The fourth domain examines clarity of guideline presentation. Recommendations must be specific, unambiguous and easily identifiable. Different options for management of the condition or health issue should be clearly presented. *Applicability* examines whether guidelines describe facilitators and barriers to application and if they provide advice and/or tools on how the recommendations can be put into practice. It also assesses resource implications for guideline application and monitoring and/or auditing criteria [18]. Transparency of guideline funding bodies and conflicts of interest were examined in *editorial independence* [18].

Each item is rated from 1 (strongly disagree) to 7 (strongly agree). Detailed criteria for each item are available within the AGREE II tool to assist the appraiser [18]. The appraisers were asked to provide comments to justify their rating. They also gave an overall assessment of the guideline from 1 (lowest) to 7 (highest) and were asked to state if they would recommend the guideline, recommend it with modifications or not recommend it.

Average appraisal scores were calculated for each appraiser by taking the average rating (1–7) for all items of a single guideline. From this, overall average appraisal scores and standard deviations were calculated for all four appraisers for a single guideline. Scaled percentages for each domain were then calculated for inter-domain comparison. This was done by adding all four appraiser ratings (1–7) of items within a single domain (obtained score) and scaling by maximum and minimum possible domain scores and converting to a percentage.

E.g.:
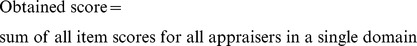


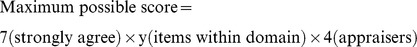


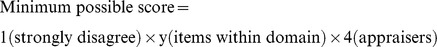


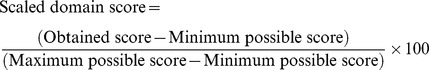
The raw appraisal scores in all four guidelines were tabulated in Microsoft Excel (Washington, USA) and sent to the appraisers for review and detection of potential rating errors. Appraisers were permitted to modify their ratings if errors were detected. Final average appraisal scores and standard deviations for each domain and scaled domain percentages were calculated. The results were shared anonymously among the authors.

## Results

Average appraisal scores and average overall assessments for each guideline are shown in Table 1. When arranged chronologically, the average overall assessment score of the quality of recommendations tended to increase over time: P-PPH 4.3 (SD: 1.0), M-PPH 5.3 (SD: 1.0), IOL 6.0 (SD: 0.0) and PE/E 6.3 (SD: 0.5). We found that the overall assessment averages were consistently higher than the average scores calculated from the individual items. All appraisers recommended the P-PPH with modifications, three recommended the M-PPH guideline with modifications and three recommended the IOL guideline without modifications. All four appraisers recommended the PE/E without modification (Table 2).

**Table 1:** Total score averages and overall assessment averages for all four guidelines from the AGREE II tool appraisals.

Guideline		Appraiser 1	Appraiser 2	Appraiser 3	Appraiser 4	Average*	SD
**P-PPH (2007)**	Average	**3.3**	**4.5**	**4.6**	**3.8**	**4.1**	**0.6**
	Overall assessment	3.0	5.0	5.0	4.0	4.3	1.0
**M-PPH (2009)**	Average	**4.2**	**5.5**	**5.3**	**4.6**	**4.9**	**0.6**
	Overall assessment	4.0	6.0	6.0	5.0	5.3	1.0
**IOL (2011)**	Average	**6.2**	**5.7**	**5.8**	**5.6**	**5.8**	**0.3**
	Overall assessment	6.0	6.0	6.0	6.0	6.0	0.0
**PE/E (2011)**	Average	**5.7**	**5.9**	**5.9**	**5.7**	**5.8**	**0.1**
	Overall assessment	6.0	6.0	7.0	6.0	6.3	0.5

* Overall average appraisal scores.

**Table 2:** Appraiser recommendations for use of guidelines.

Guideline	Assessor 1	Assessor 2	Assessor 3	Assessor 4
**P-PPH (2007)**	Recommended, with modifications	Recommended, with modifications	Recommended, with modifications	Recommended, with modifications
**M-PPH(2009)**	Recommended, with modifications	Recommended	Recommended, with modifications	Recommended, with modifications
**IOL (2011)**	Recommended	Recommended	Recommended, with modifications	Recommended
**PE/E (2011)**	Recommended	Recommended	Recommended	Recommended

Table 3 presents the scaled domain percentages for all four guidelines. Concerning *scope and purpose* of the guidelines, the recent guidelines scored highest, though since 2007 the scores were relatively high (79%). *Stakeholder involvement* was rated higher over time - P-PPH (2007) scored 32%, M-PPH (2009) 51%, IOL (2011) 69% and PE/E (2011) 86%. *Rigour of Development* scores tended to be higher in the recent guidelines, although scores were relatively high in all four guidelines. *Clarity of Presentation* scored over 90% in the IOL and PE/E guidelines. The appraisers gave relatively low scores on *applicability* throughout all four guidelines, although the P-PPH from 2007 scored the lowest with 22%, followed by M-PPH, scoring 29% and IOL and PE/E scoring 61% and 58%, respectively. *Editorial independence* has the most variation in scores as presented in the table. The complete assessments of all four appraisers are presented in Tables S1, S2, S3, S4, S5, S6, S7 and S8.

**Table 3:** Scaled domain percentages for all appraisers for each guideline.

Domain	P-PPH (2007)	M-PPH (2009)	IOL (2011)	PE/E (2011)
**Scope and purpose (%)**	79	68	90	89
**Stakeholder involvement (%)**	32	51	69	86
**Rigour of development (%)**	66	79	88	84
**Clarity of presentation (%)**	71	83	97	93
**Applicability (%)**	22	29	61	58
**Editorial independence (%)**	8	71	60	65

## Discussion

We used the AGREE II online guideline assessment tool to evaluate the quality of four WHO reproductive health guidelines issued between 2007 and 2011 [13]–[16]. The AGREE II tool assesses several guideline domains and evaluates their quality using numeric scores (higher scores suggest a higher quality of the respective domain) (REF). In general, the two most recent guidelines (2011) tended to receive higher AGREE II scores as compared to the guidelines issued in 2007 and 2009. This may suggest an improvement in the quality of those guidelines according to the AGREE methods.

It should be noted that among the six domains evaluated by the AGREE II tool, three of them (Scope and purpose, rigour of development and clarity of presentation) had received scores in the higher end of the spectrum of quality in all four guidelines. This may be due to the fact that the WHO Department of Reproductive Health and Research is recognized as having a very strong methodological component and a large experience in randomized trials and systematic reviews.

However, the involvement of stakeholders, particularly the integration of the *“views and preferences of the target population”*, remains a challenge. RHL commentators conducting independent assessments of these guidelines have also identified this as a weakness of some of these guidelines [21]. The WHO Guideline Review Committee suggests that the views of end-users and patients are considered during the development of WHO Guidelines [22]. It is ethically worthwhile to include consumer representation in the development process and to acknowledge their views and values where possible. Nevertheless, although desirable, consumer representation and engagement may be not straightforward in guidelines (such the ones produced by WHO) that target many diverse settings in low and middle-income countries.

The appraisers identified clarity and visibility of key recommendations as strengths of the IOL and PE/E guidelines. However, appraisers suggested that presenting the guideline in two versions would be of benefit: one containing a detailed description of methodology and evidence, and a simpler version with key messages only. This would allow target users to capture the key recommendations easily, improving compliance with best practice.

*Applicability* and *editorial independence* were the lowest scoring domains in the two most recent guidelines. The low scores in the *applicability* domain (61% and 58% for IOL and PE/E respectively) reflect poor scoring in items on resource implications and cost effectiveness. However, WHO guidelines target a wide variety of countries, making specific and detailed information about resources and financial costs problematic. The RHL commentators drew similar conclusions for the IOL guideline, remarking that while no cost analysis was conducted, the recommendations are “feasible in under-resourced settings” and “likely to be cost–effective and acceptable to the pregnant women, their obstetricians and policy-makers in under-resourced settings” [21]. Whether economic assessments are necessary or feasible for global guidelines might indicate a contextuality issue in applying the AGREE II tool to WHO guidelines.

In spite of the fact that the funding bodies of these four guidelines are essentially governmental agencies and academic institutions (without commercial interests in the content of the recommendations), appraisers noted that additional details on the role of the *funding bodies in the content of the guideline* would be of benefit. Disclosure of funding sources and influence contributes to a more transparent process and is in line with existing WHO policy.

Oxman et al. (2007) pointed out several specific weaknesses of the WHO guideline development process. They highlighted an absence of systematic, transparent methods of synthesizing and presenting evidence, as well as infrequent use of systematic reviews and over-reliance on expert opinion [3]. Using the AGREE methodology, the domains *rigour of development* and *scope and purpose* had higher scores in the most recent guidelines. This may suggest possible improvements in defining objectives and target populations as well as in systematic literature searching methodologies and transparency of the evidence-gathering process. Oxman et al. (2007) also identified a lack of timelines for updating and plans for dissemination and implementation [3]. The appraisers tended to rate higher the more recent guidelines on documented updating strategies within the *rigour of development* domain. The implementation and dissemination aspects of guidelines received also higher scores in the most recent guidelines.

There are limitations within our analysis that should be noted. The AGREE II tool has been tested for reliability and validity, and is applicable to a wide variety of health professionals, geographical areas and guideline development processes [23]–[25]. However, there is no threshold for discriminating “high quality” from “low quality” guidelines, leaving appraisers to interpret scores. Thus, the scores of an AGREE evaluation have to be interpreted with caution and in context. Furthermore, no reliable statistical conclusions can be drawn from a small number of appraisers assessing guidelines in a semi-quantitative manner. In this case study, a small number of guidelines that have been produced sequentially over time, is evaluated. Considering the small number of guidelines (only 4), it cannot be ruled out that the changes in the scores are due to chance. However, it is plausible that the growing experience of guideline development processes (particularly after the establishment of the WHO Guideline Review Committee in 2007, which may have led to an increased awareness of quality and transparency in the guideline development) could have contributed to scores that tended to be higher in the most recent guidelines.

Appraisers also remarked that if a guideline is presented in a more structured way (without improvements in content), this would lead to a more positive evaluation overall. Another limitation is that the appraisers were health professionals relatively inexperienced in guideline development and evaluation and not blinded to the publication year of the guidelines they appraised. At the time of appraisal they were on temporary, voluntary assignment in RHR, which may constitute a potential conflict of interest that needs to be considered. These factors may have affected the quality of results and may have been a potential source of bias. However, we tried to minimize bias ensuring adequate training on the use of the AGREE II tool before the actual guideline appraisal. It is worthwhile noting that the same four appraisers assessed all four guidelines using the AGREE II tool. Anonymisation of results and instructions to not communicate during the appraisal process may also have contributed to reducing the potential bias. With these actions, conditions for effective use of the evaluating tool and a meaningful inter-appraiser comparison were fostered. In addition, notwithstanding having no participation in the guideline assessment, three of the authors (JPS, MM, AMG) are WHO employees and have been involved in the development of the guidelines under assessment.

In conclusion, the appraisals suggest that the process of guideline development and quality of reporting are robust in maternal and perinatal health guidelines produced by WHO. Among the remaining challenges, the involvement of stakeholders and the applicability aspects should be highlighted. Considering the large number of guidelines produced by WHO, findings of this assessment may be indicative of change, but a more comprehensive assessment is needed in order to demonstrate or not a change in the process of guideline development and the quality of reporting in WHO.

## Supporting Information

Table S1 **Assessment of the Guidelines on Prevention of PPH.**
(XLSX)

Table S2 **Assessment of the Guidelines on Management PPH and retained placenta.**
(XLSX)

Table S3 **Assessment of the Guidelines on Induction of labour.**
(XLSX)

Table S4 **Assessment of the Guidelines on Eclampsia.**
(XLSX)

Table S5 **Total score averages and overall assessment averages for all four guidelines.**
(XLSX)

Table S6 **Sums and Percentages.**
(XLSX)

Table S7 **Scaled domain percentages for all appraisers for each guideline.**
(XLSX)

Table S8 **Appraiser recommendations for use of guidelines.**
(XLSX)
